# [^18^F]FDG PET-MR in the Evaluation and Follow-Up of Incidental Bone Ischemic Lesions in a Mono-Center Cohort of Pediatric Patients Affected by Hodgkin’s Lymphoma

**DOI:** 10.3390/diagnostics13030565

**Published:** 2023-02-03

**Authors:** Chiara Giraudo, Elisa Carraro, Elena Cavallaro, Monica Zuliani, Liliya Spampinato Gotsyak, Davide Massano, Antonella Modugno, Lara Mussolin, Alessandra Biffi, Diego Cecchin, Marta Pillon, Pietro Zucchetta

**Affiliations:** 1 Department of Medicine-DIMED, Nuclear Medicine Unit, Padova University Hospital, 35128 Padova, Italy; 2 Pediatric Hematology, Oncology and Stem Cell Transplant Division, Padova University Hospital, 35128 Padova, Italy; 3 Maternal and Child Health Department, Padova University, 35128 Padova, Italy; 4 Department of Medicine, Pediatric Radiology Unit-Neuroradiology Institute Hospital, University of Padova, 35128 Padova, Italy; 5 Radiology Unit, Hospital of Camposampiero, 35012 Camposampiero, Italy; 6 Istituto di Ricerca Pediatrica Città della Speranza, 35127 Padova, Italy; 7 Department of Medicine-DIMED, Nuclear Medicine Unit, School of Medicine, University Hospital of Padova, 35128 Padova, Italy

**Keywords:** bone ischemic lesion, childhood, adolescent, PET-MR, Hodgkin’s lymphoma

## Abstract

Hodgkin’s lymphoma (HL) is one of the neoplasms with the best prognosis in children, adolescents and young adults, but sufferers are burdened by the possibility of developing adverse effects such as Bone Ischemic Lesions (BILs) which are lesions of the bone caused by the loss of/reduction in blood flow. The main goal of this retrospective study was to evaluate the role of [^18^F]FDG-PET-MR in the early detection of BILs in a single-center cohort of uniformly treated pediatric HL patients. BILs were assessed through PET-MR images as the appearance of medullary lesion surrounded by a serpiginous, tortuous border. From 2017 to 2022, 10/53 (18.9%) HL patients developed BILs which were mostly (8/10 patients) multifocal. Overall, 30 lesions were identified in the 10 asymptomatic patients, all with the above-mentioned features at MR and with very low [^18^F]FDG uptake. BILs were incidentally detected during HL therapy (*n* = 6) and follow-up (*n* = 4), especially in the long bones (66.7%). No factors correlated with the occurrence of BIL were identified. No patients developed complications. PET-MR is a sensitive combined-imaging technique for detecting BILs that are asymptomatic and self-limiting micro-ischemic lesions. BILs can be monitored by clinical follow-up alone both during and after therapy.

## 1. Introduction

Hodgkin’s Lymphoma (HL) is one of the most frequent cancers among children, adolescents and young adults (CAYAs) [1,2,3]. In the last 30 years, collaboration among cooperative study groups allowed a 5-year overall survival rate higher than 90% to be achieved despite a higher increased risk of early mortality and long-term sequelae [1,2,3,4,5].

Recently, the European Network for Pediatric Hodgkin Lymphoma (EuroNet-PHL) group designed the international EuroNet-PHL C2 trial (EudraCT-Number: 2012-004053-88) for the treatment of classic HL in CAYAs with the aim of avoiding radiotherapy and its subsequent effects. The EuroNet-PHL-C2 protocol includes the imaging evaluation of therapy response at two time points over the treatment according to the whole body (WB) 2-[^18^F]fluoro-2-deoxy-D-glucose (FDG) positron emission tomography (PET) scan. As a standard of care, PET could also be used to investigate the treatment response in the case of refractory or relapsed disease. PET could be combined with Computed Tomography (CT) or with Magnetic Resonance (MR) imaging scans in one imaging session [6]. Recently, Cistaro et al. showed the high diagnostic value of FDG-PET-CT for the assessment of bone marrow (BM) involvement in CAYAs affected by HL instead of the BM biopsy [7]. PET-MR, when available, is preferred over PET-CT, mostly for the better evaluation of soft tissue and BM involvement. In addition, at diagnosis or during response assessment, PET-MR could highlight bone alterations which were not necessarily related to HL and were consistent with ischemic damages, including BILs—a condition that results from a reduction in or complete loss of blood supply to a bone region [8]. Available data about the real incidence and prognosis of BILs are scarce because prospective WB-MR studies are lacking in CAYAs with cancer, in contrast with the numerous published data on ischemic lesions [9,10]. In particular, most of the data resulted from studies of pediatric cancer survivors, selected case series or investigations performed on symptomatic patients [6,11]. The only prospective study which evaluated ischemic lesions in a cohort of pediatric HL patients during and after uniform treatment by WB-MR reported on 10 patients (41.7%, 95% confidence interval: 22–61%) with a total of 56 areas of ON without signs of bone collapse. In view of these data, the authors suggest that the detection of early ON could allow for preventative treatment before bone or joint damage occurs [10].

The main aim of this study was to evaluate by retrospective analysis the diagnostic role of PET-MR in the early detection of BILs in a single-center cohort of pediatric HL patients uniformly treated according to the EuroNet-PHL-C2 protocol. Secondary objectives were the analysis of possible predisposing factors for the development of BILs and their clinical impact. 

## 2. Materials and Methods

### 2.1. Study Design

This was a retrospective, mono-center study. Patients less than 18 years old, newly diagnosed with classic HL, were enrolled from March 2017 to November 2021 in the EuroNet-PHL-C2 protocol and were followed for a minimum of six months after stop therapy. The study was carried out at the Pediatric Oncohematology Clinic-Department of Women’s and Children’s Health, University of Padua, Italy, and was conducted in compliance with Good Clinical Practice (GCP) guidelines. The retrospective study was notified to the local Ethics Committee. The inclusion criteria were as follows: histologic confirmation of classic HL; age at diagnosis less than 18 years; treatment according to the EuroNet-PHL-C2 protocol (NCT02684708); disease assessment by PET-MR at diagnosis and at least at first disease re-evaluation after 2 chemotherapy courses; negative pregnancy test for females; ongoing follow-up in the same facility. Previous chemotherapy or radiation treatment for other cancers and severe concomitant diseases were considered as exclusion criteria. Patients were treated according to the treatment level (TL) expected by the EuroNet-PHL-C2 protocol. At the end of the two induction cycles with Vincristine, Etoposide, Prednisone and Doxorubicin (OEPA), all patients underwent the scheduled re-evaluation PET-MR assessment (ERA-PET). In cases of inadequate response to therapy, patients performed a late re-evaluation PET-MR assessment (LRA-PET) at the end of chemotherapy. In cases of doubtful imaging, recurrence or disease progression, further PET-MR evaluations were performed as standard of care during follow-up. 

### 2.2. PET-MR Protocol and Image Analysis

**Figure 1:**
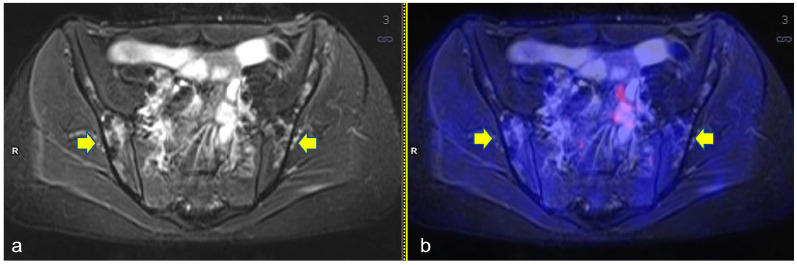
Axial turbo inversion recovery magnitude (TIRM) image (**a**) and fused axial TIRM and [^18^F]FDG-positron emission tomography (PET) (**b**) of the PET/MR scan of a 17 year-old girl affected by Hodgkin’s lymphoma, performed for re-evaluation after chemotherapy (ERA-PET), clearly demonstrating the presence of ischemic lesions in the iliac bones (yellow arrows), with no [^18^F]FDG uptake.

The [^18^F]FDG-PET-MR was performed with an integrated, simultaneous, hybrid PET-MR device (Biograph mMR; Siemens, Erlangen, Germany) operating a 3T, with high performance gradient systems (45 mT/m) and equipped with a phased-array body coil. The scan was performed following the SNMMI/EANM guideline [12]. Briefly, the images (5 min/bed position) were acquired 60 min after intravenous injection of [^18^F]FDG (≥6 h fasting, 3 MBq/kg), from the vertex to toes. The MR protocol included, in addition to the axial and coronal high-resolution and breath-hold CAIPIRINHA for attenuation correction and anatomic correlation, whole-body axial Turbo Inversion Recovery Magnitude sequence (TIRM) (inversion time 220 ms, repetition time (RT)/echo time (ET) 6860/76 ms, and slice thickness 4 mm) and whole-body axial HASTE (RT/ET 1600/95 ms, slice thickness 5 mm). The total scanning time for each patient was about 45 min, depending on the patient’s height. One nuclear medicine physician and one radiologist with expertise in pediatric oncological imaging revised all PET-MR scans of each patient in consensus, reporting the occurrence of ischemic skeletal lesions, defined as serpiginous areas of hyperintensity on axial TIRM surrounded by areas of hypointense sclerosis on all sequences C (Figure 1). The number of lesions, affected bone/s and the presence of metabolic activity (using the mediastinal blood pool as a reference) were recorded for each patient.

### 2.3. Data Analysis

Data were retrospectively collected by direct, anonymous consultation of patients’ medical records. In particular, the data were analyzed as follows: gender; Body Mass Index (BMI); age at diagnosis; performance of competitive sports activity; histologic variant of HL; stage of disease at diagnosis; presence of bulky mass; presence of B symptoms (weight loss > 10% in the 6 months prior to diagnosis, night sweats, fever); site of disease; level of treatment; adequate or inadequate response to PET-MR re-evaluations; occurrence of disease progression or relapse; development of BILs detected by PET-MR imaging investigations; presence of thrombosis during chemotherapy. A descriptive analysis was conducted on the entire population. Quantitative variables were expressed as a mean, median, range or percentage value. Qualitative variables were described as a present or absent characteristic. Chi-square test or Fisher’s exact test for discrete variables and Student’s *t*-test for continuous variables were used to compare patients with or without BILs. Differences between the parameters were considered statistically significant with a *p*-value < 0.05. Statistical analyses were performed using SAS statistical software (SASPC, version 9.4, SAS institute, Cary, NC, USA).

## 3. Results

### 3.1. Patients

Among the 56 patients registered, three were not eligible due to second cancer (*n* = 1) or comorbidities (*n* = 2). Overall, 53 patients affected by classical HL were enrolled in this study; 94.3% of them presented a nodular sclerosis histological subtype (*n* = 50) and the remaining patients had a lymphocyte-rich (*n* = 1) or mixed cellularity subtype (*n* = 2). According to the Ann Arbor Classification staging disease, 31 patients were in stage II, 10 in stage III and 12 in stage IV [13]. Median age at diagnosis was 15.1 years (range 8.2–17.9 years) and the median follow-up was 2.0 years (range 0.5–4.8 years). B-symptoms were present in 14 (26.4%) patients at diagnosis. All patients had nodal disease, while 18 had also extranodal involvement, including the bone site in 6 patients. They were treated according to the TL1 (*n* = 5), TL2 (*n* = 26) or TL3 (*n* = 22) of the EuroNet-PHL-C2 international protocol. The disease re-evaluations after two OEPA courses showed 36 (68%) patients with an adequate response, while the remaining 15 patients had an inadequate response. Three patients relapsed after the end of therapy. Overall, 10 patients underwent RT. The vast majority of patients were a normal weight (91%), four were considered overweight and only one patient was obese. Before starting cancer chemotherapy, 12 patients were actively participating in competitive sports (Table 1).

### 3.2. Bone Ischemic Lesions

**Figure 2:**
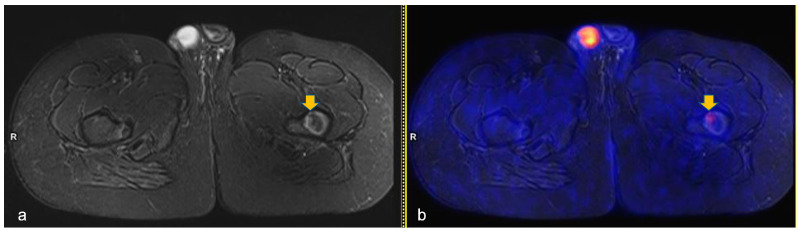
Axial turbo-inversion recovery magnitude (TIRM) image (**a**) and fused axial TIRM and [^18^F]FDG-positron emission tomography (PET) (**b**) of the PET/MR scan of a 13 year-old boy affected by Hodgkin’s lymphoma, performed for re-evaluation after chemotherapy (ERA-PET), clearly demonstrating the presence of an ischemic lesion in the left femur (orange arrow in (**a**)) with mild, focal uptake (SUVmax 2.4, orange arrow in (**b**)).

A total of 141 PET-MR were analyzed: 53 scans were performed at diagnosis and the same number at the first scheduled evaluation (ERA-PET-RM), 15 at the late evaluation (LRA-PET-RM) and 20 were performed during follow-up. Overall, 30 BILs were detected in 10 out of 53 patients (18.9%). All lesions showed very low [^18^F]FDG uptake (standardized uptake value-SUVmax mean 1.6, Stand Dev. 0.5, min 0.3 max 2.2) and only one lesion was above SUVmax 2 (Figure 2). 

Patients’ characteristics according to the presence of BILs are reported in Table 1. According to the BMI, one patient was categorized as overweight and one other patient as obese with altered lipid balance. Hormone assays were performed on all patients, and no abnormalities were found. Two out of 10 patients were prepubertal, and the remaining pubertal. No statistically significant differences were observed among patients with or without BILs according to the prognostic factors analyzed (Table 1). The sites and the timing of the BILs are illustrated in Table 2.

**Figure 3:**
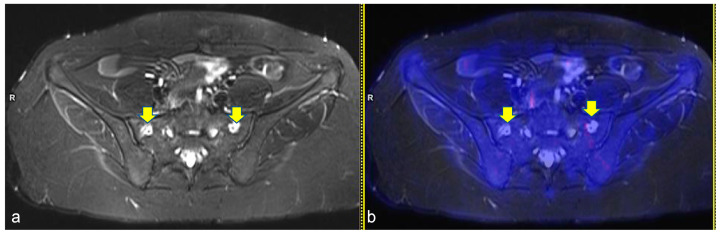
Axial turbo inversion recovery magnitude (TIRM) image (**a**) and fused axial TIRM and [^18^F]FDG-positron emission tomography (PET) (**b**) of the PET/MR scan of a 17 year-old girl affected by Hodgkin’s lymphoma, performed for re-evaluation after chemotherapy (ERA-PET), clearly demonstrating the presence of symmetric ischemic lesions in the sacrum (yellow arrows in (**a**)) without pathologic uptake.

The sites most affected by BILs were the long bones (*n* = 20, 66.7%), particularly the proximal humerus, the proximal (Figure 2) and distal femur and the proximal tibia. A total of 80% (*n* = 16) of the BILs of the long bones were detected in the lower limbs. The remaining 4 lesions of the upper limbs were described only in the proximal humerus; no involvement of the distal humerus was observed. In the lower limbs, there were 10 lesions in the proximal femur, 3 lesions in the distal femur and 3 lesions in the proximal tibia. Less frequent BILs’ localizations were the pelvis (*n* = 4) (Figure 3), the scapula (*n* = 1) and the vertebrae (*n* = 1). Overall, 80% of the patients (*n* = 8) with BILs had a total of 28 multifocal localizations: two sites in three patients, four sites in three patients, and five sites in two patients. Only two patients had isolated BILs: one in the right iliac bone and one in the left distal femoral diaphysis. Overall, 10 lesions were detected in the femural (*n* = 6), humeral (*n* = 3) and tibial (*n* = 1) epiphysis. No BILs were detected at staging, including in the two patients with bone involvement of HL at diagnosis. Overall, 60% of BILs were described at the planned ERA PET-RM evaluation after two OEPA cycles (median time from diagnosis: 53 days, range 50–56 days), whereas the remaining 40% were reported during PET assessment for disease follow-up (median time from diagnosis: 8 months, range, 6–11 months). In four patients who performed further PET-MR as clinical follow-up, after BILs detection, the lesions remained unchanged. During the patients’ clinical follow-up (median time: 2.4 years, range 1–4.4 years), all of them had resumed normal daily and physical activities and one patient had restarted athletic agonistic activity, without developing symptoms such as pain or reduced mobility. 

## 4. Discussion

To our knowledge, this is the first study and, overall, largest study demonstrating the occurrence and the incidence of BILs using [^18^F]FDG-PET-MR in a cohort of CAYAs with classic HL uniformly treated for their hematological disease. Indeed, Theruvath et al. previously reported such evidence in a cohort of 10 oncological patients with different types of tumors (e.g., non-HL, HL, rhabdomyosarcoma, and leukemia) [6]. The paucity of evidence regarding this finding might be due to the low number of PET-MR scanners in European centers [14,15,16,17]. Up to now, few data are available regarding the incidence of BILs since they are frequently discovered fortuitously during imaging assessment for other reasons [18]. BILs represent incidental findings during disease evaluation in patients without bone and musculoskeletal pain symptoms as presented in our case series [18,19]. For these reasons, comparison with the existing literature is challenging, especially since these are often publications on case reports or case histories of ON disease [6,9]. 

In our study, it was possible to identify an incidence of approximately 19% of patients developing bone lesions during and after therapy for lymphoma in a cohort of 53 pediatric patients, which is much lower than the 41% incidence of ON that was demonstrated by Littooij et al. in a cohort of only 24 pediatric patients with HL studied during and after therapy by WB-MR scanning [10]. Moreover, a prospective pilot study focused on the radiological findings assessed by PET-MR after stop therapy in pediatric cancer survivors found 25 osteonecrotic lesions in 50% of the 10 enrolled patients [6]. In light of these data, it can be hypothesized that the detection of BILs observed in our case history may represent an early detection of ischemic damage that needs to be monitored to establish its evolution, if any, and may become a well-known subsequent effect in the same way as the frequently observed ON in pediatric patients affected by acute lymphoblastic leukemia (ALL) [9]. 

On PET images the BILs showed low uptake due to having low metabolic activity, and on MR images they appeared as serpiginous areas with a low signal on T1W and a hyperintense signal on T2W images due to granulation tissue, surrounded by areas of hypointense sclerosis on all sequences [20]. In our case series, the 30 observed BILs presented the particular combined features that are common in the manifestation of ON [8]. In our study, it is of note that 66.7% of the BILs were detected in the meta-diaphysis of long bones, unlikely according to the literature which reported the majority of ON in epiphysis and rarely in metaphysis [8,10,20]. In addition, the literature described ischemic–necrotic lesions of bones frequently accompanied by symptoms such as pain and reduced joint mobility, with debilitating degenerative patterns that can evolve into joint collapse and the need for prosthetic replacement [21]. In our study all patients, despite five having epiphyseal lesions, were asymptomatic at the onset of BILs and throughout the clinical follow-up. Moreover, the lesions remained stable during the follow-up in patients who underwent further PET-MR investigations. Considering a median follow-up of over 2 years and that all 10 patients affected by BILs resumed their sport activities without any limitation, it can be assumed that BILs are self-limiting lesions, at least in the short-term clinical follow-up. As a consequence, BILs were not further investigated by WB-MR to assess the progression of the lesions, and bone imaging re-evaluation was suggested as part of the follow-up program for cancer survivors. In cases of pain occurrence related to the initially investigated BILs, it is of note that additional WB-MR would be recommended to confirm possible evolution of the skeletal lesions. In the presence of worsened lesions, patients should be enrolled in a conservative, rehabilitation program.

The present results are consistent with the literature as the most commonly involved sites were the long bones (67%), particularly the proximal humerus, the proximal and distal femur and the proximal tibia [20]. In fact, the subjects enrolled in this study were all in the pediatric–adolescent age group and thus in the phase of bone growth, a process that occurs from the growth plaques of the long bones which are therefore more susceptible to the development of bone injury. BILs are lesions with a tendency for multifocality, as confirmed by our case series where 80% of patients developed more than one lesion simultaneously, showing 50% of patients with at least three concomitant lesions [22]. 

Patients’ characteristics in the whole study group are in line with the reported characteristics’ distribution for CAYA patients affected by classic HL, demonstrating a not-selected population despite the retrospective nature of the study [1,2,3,4,5]. In order to investigate possible predisposing factors for BILs in this HL cohort, the main and well-known predisposing factors for pediatric CAYA patients affected by ALL, such as age, sex, alterations in lipid balance, BMI and thrombosis, were included in the analysis [20,22]. No significant prognostic factors emerged in our study or in the results reported by Littooij who also studied a pediatric cohort of patients with HL [10]. It is of note that predominance was observed at the time of onset of BILs in the present study. No analysis regarding the corticosteroids’ doses was reported since a section of the patients included in this study was enrolled in the EuroNet PHL-C2 trial for which results are not yet available. Interestingly, two of the patients who developed BILs had bone involvement in the staging phase of the hematological disease and in one case the development of BILs affected the same sites as the disease in the bone. In fact, given the typical features at MR imaging and the slightest pathological tracer uptake, PET-MR allows a precise distinction between BILs and neoplastic marrow infiltration. The small number of patients does not allow a firm conclusion to be drawn, but it seems reasonable to consider any uptake as probably benign at least up to the threshold of SUV = 2.

Prospective studies may allow the evaluation of additional prognostic factors in a series of consecutively enrolled patients, reducing the possible bias related to retrospective studies. A longer follow-up period, based on a scheduled evaluation program for cancer survivors, will allow a more precise definition of BILs and their possible evolution. In particular, given the overall low metabolic activity of BILs, probably MR or WB-MR in the case of multiple lesions could be sufficient to monitor their changes over time.

Moreover, further projects should investigate the role of Diffusion Weighted Imaging and in particular the correlation between Apparent Diffusion Coefficient values and the metabolic activity in BILs. In fact, several studies already demonstrated the additional value provided by this sequence for ischemic skeletal lesions such as avascular necrosis of various etiologies [23,24,25]. Unfortunately, this type of analysis was not feasible in our study because this sequence was only available for a few of the children examined. Lastly, further studies may investigate the role of other additional quantitative MR imaging techniques for BILs, such as T1 and T2 mapping or bone perfusion. In fact, Johnson and colleagues used an animal model to demonstrate that relaxation time-mapping can detect hip ischemia in a developing femoral head while Cultot et al. showed that in patients with osteonecrosis of the femoral head, vascular phenomena beyond the necrotic area prevail [26,27].

Monitoring of BILs in CAYA patients with HL should be included as part of the follow-up program for cancer survivors. This will allow the detection of possible bone damage and will allow for a rapid and personalized intervention to be planned, when necessary, in order to ensure the patient’s quality of life.

## 5. Conclusions

PET-MR is a sensitive, combined imaging technique when used for detecting BILs that are micro-ischemic lesions characterized by no symptoms and a self-limiting behavior in pediatric HL. BILs can be observed as incidental findings during and after therapy for HL. BILs can be monitored by clinical follow-up alone during and after therapy. Future researchers are needed to investigate additional predisposing factors and to identify tailored follow-up programs.

## Figures and Tables

**Table 1:** Patients’ characteristics at HL diagnosis according to the bone ischemic lesion (BILs).

		Patients with BILs		Patients without BILs		*p*-Value
		N	%	N	%	
Gender	Male	6	60%	14	33%	0.15
	Female	4	40%	29	67%	
Age at diagnosis	Median	16.3		15		0.45
	Range	9.4–17.9		9.4–17.9		
Body mass index	Normal	8	80%	40	93%	0.43
	Overweight	1	10%	3	7%	
	Obese	1	10%	0	0%	
Agonistic sport	Yes	1	10%	11	26%	0.42
	No	9	90%	32	74%	
	I [A/B]	0	0%	0	0%	0.79
Stage	II [A/B]	5	50%	26	60%	
	III [A/B]	2	20%	8	19%	
	IV [A/B]	3	30%	9	21%	
	TL1	0	0%	5	11%	0.92
Treatment level	TL2	5	50%	21	49%	
	TL3	5	50%	17	40%	
	Lymph node	10	100%	43	100%	1.0
	Spleen	1	10%	10	23%	
Primary site of disease	Extra-lymphatic	5	50%	13	30%	
	Lung	2	20%	8	19%	
	Liver	1	10%	1	2%	
	Bone	2	20%	4	9%	
B symptoms	Yes	2	20%	12	28%	1.0
	No	8	80%	31	72%	
	Scleronodular	9	90%	41	95%	0.45
Histological subtype	Lymphocyte-rich	1	10%	0	0%	
	Mixed cellularity	0	0%	2	5%	
Bulky disease	Yes	5	50%	17	40%	0.72
	No	5	50%	26	60%	
Disease involvement	Yes	2	20%	4	9%	0.32
	No	8	80%	39	91%	
Thrombosis	Yes	0	0%	4	9%	1.0
	No	10	100%	39	91%	
Event	Yes	1	10%	2	5%	0.47
	No	9	90%	41	95%	
Radiotherapy	Yes	4	40%	6	14%	0.19
	No	6	60%	36	84%	
AR at ERA-PET-MR	Yes	6	60%	32	74%	0.27
	No	4	40%	11	26%	

Event defined as disease progression or recurrence of disease; AR: adequate response.

**Table 2:** Clinical characteristics of the 10 patients with bone ischemic lesion.

Pts	TL	HL Disease Bone Involvement at Diagnosis	BILs Site Involvement	N. of Lesions	PET-MR Time-Point
1	3	Yes	bilateral distal femur diaphysis; right proximal tibia metaphysis and left proximal tibia diaphysis	4	ERA PET-MR
2	2	No	right iliac wing; proximal humerus epiphysis; bilateral proximal femurs epiphysis	5	ERA PET-MR
3	3	No	right iliac wing; sacrum; bilateral proximal femoral metaphysis	4	ERA PET-MR
4	3	No	left proximal humerus epiphysis; iliac wings; S2 vertebrae	5	Follow-up (+9 months from diagnosis)
5	2	No	right iliac wing	1	Follow-up (+7 months from diagnosis)
6	3	Yes	left proximal tibia epiphysis; left proximal femur diaphysis	2	ERA PET-MR
7	2	No	right proximal humerus epiphysis; bilateral proximal femur epiphysis; right scapula	4	Follow-up (+6 months from diagnosis)
8	2	No	left distal femoral diaphysis	1	ERA PET-MR
9	2	No	mid-proximal left femur diaphysis; left proximal humerus metaphysis	2	ERA PET-MR
10	3	No	bilateral proximal femur epiphysis	2	Follow-up (11 months from diagnosis)

## Data Availability

The data presented in this study are available on request from the corresponding author.
